# Clinical and pathological findings of SARS-CoV-2 infection and concurrent IgA nephropathy: a case report

**DOI:** 10.1186/s12882-020-02163-3

**Published:** 2020-11-24

**Authors:** Yi Huang, Xiao-Juan Li, Yue-Qiang Li, Wei Dai, Tiffany Shao, Wei-Yong Liu, Min Han, Gang Xu, Liu Liu

**Affiliations:** 1 Department of Nephrology, Tongji Hospital, Tongji Medical College, Huazhong University of Science and Technology, 1095 Jiefang Avenue, Wuhan, 430030 Hubei P.R. China; 2 Department of Pathology & Laboratory Medicine, University of Calgary, Alberta Precision Laboratories, Calgary, AB Canada; 3 Department of Laboratory Medicine, Tongji Hospital, Tongji Medical College, Huazhong University of Science and Technology, Wuhan, China

**Keywords:** COVID-19, IgA nephropathy, Case report, Macroscopic hematuria, Acute kidney injury

## Abstract

**Background:**

Since the Coronavirus Disease 2019 (COVID-19) outbreak, there is accumulating data on the clinical characteristics, treatment strategies and prognosis of COVID-19 in patients with concurrent renal disease. Postmortem investigations reveal renal involvement in COVID-19, and most recently, several biopsy researches reveal that acute tubular injury, as well as glomerular nephropathy such as collapsing glomerulopathy were common histological findings. However, to our best knowledge, there is limited data regarding IgA nephropathy in the setting of COVID-19.

**Case presentation:**

In the present case, we report a 65-year old Chinese woman who presented with dark-colored urine, worsening proteinuria and decreased renal function after COVID-19 infection. She received a renal biopsy during COVID-19 infection. The renal biopsy revealed IgA nephropathy without any evidence for SARS-Cov-2. The findings suggest that the renal abnormalities were a consequence of exacerbation of this patient’s underlying glomerular disease after COVID-19 infection. After a regimen of 3-day course of glucocorticoid and angiotensin II receptor blocker therapy, the patient recovered and remained stable upon follow-up.

**Conclusions:**

It is important to consider the underlying glomerular disease exacerbation as well as virus induced injury when dealing with renal abnormalities in patients with COVID-19. A kidney biopsy may be indicated to exclude a rapidly progressive glomerular disease.

## Background

The Coronavirus Disease 2019 (COVID-19) is an emerging infectious disease attributed to the infection by a novel coronavirus termed severe acute respiratory syndrome coronavirus 2 (SARS-Cov-2) [1]. Chronic kidney disease (CKD) that accounts for 0.7–2.9% of the investigated population is not a frequent underlying condition in patients with COVID-19 [2–4]. Patients in older age and with comorbidities such as hypertension are at a higher risk to progress. Currently, there is no robust evidence to indicate that patients with CKD are at an increased risk compared with other comorbidities. To date, publications linking COVID-19 with renal comorbidities are mostly focused on patients with end-stage renal disease [5, 6]. IgA nephropathy is the most common primary glomerular disease [7], and the impact of COVID-19 on patients with glomerular diseases has not been studied. In the current report, we present the clinical course and histological findings in a patient with underlying IgA nephropathy who infected with SARS-CoV-2.

## Case presentation

The patient is a 65-year-old Chinese woman with a 4-year history of hypertension, and a 14 months history of proteinuria and microscopic hematuria. The patient had been in her baseline renal condition until January 9, 2020. As shown in Table 1, her baseline estimated glomerular filtration rate (eGFR) in the past year range from 64.2 ml/min/1.73m^2^ to 72.6 ml/min/1.73m^2^. Baseline urine sediment examination showed 36.61 cells/μl − 74.43 cells/μl in erythrocyte count and baseline proteinuria excretion was up to 510 mg/day. Three days prior to her admission, she got flu-like symptoms including headache, myalgia and fatigue, which were resolved in 1 to 2 days. However, she developed dark-colored urine and flank pain a day later and presented to the out-patient clinic. Urine sediment investigation showed significant worsening in urine erythrocyte count (2518.03/μL) and she was admitted to the hospital on January 10, 2020. On admission, vital signs were normal with a body temperature of 36.8 °C, blood pressure of 149/104 mmHg, heart rate of 80 beats per minute, and a respiratory rate of 16 breaths per minute. Both lungs were clear to auscultation. The rest of the physical examination was also unremarkable.

**Table 1:** Laboratory characteristics of the patient

Date	Out-patient				Clinic	Hospital		Discharge	Follow-up	Reference range
	2019.1.25	2019.3.18	2019.5.22	2019.9.23	2020.1.9	2020.1.10	2020.1.20	2020.1.25	2020.4.20	
**RBC, cells/μl**	70.4	NA	NA	36.6	2518.0	98.9	NA	NA	28.3	0–30
**Proteinuria excretion, grams/24 h**	0.51	0.45	0.27	0.43	NA	1.07	NA	NA	NA	≤140
**Creatinine,** μmol**/L**	83	75	80	75	84	96	74	79	73	45–84
**eGFR, ml/min/1.73m**^**2**^	64.2	72.6	67.1	72.1	62.8	53.6	73.5	67.9	74.7	> 90
**UACR, mg/g**	43.6	NA	NA	36.1	NA	288.8	NA	NA	33.61	0–30

*RBC* urine red blood cell count, *WBC* blood white blood cell, *eGFR* estimated glomerular filtration rate, *hs-CRP* hyper-sensitive C reaction protein, *UACR* urine albumin to creatinine ratio, *NA* not applied

**Fig. 1:**
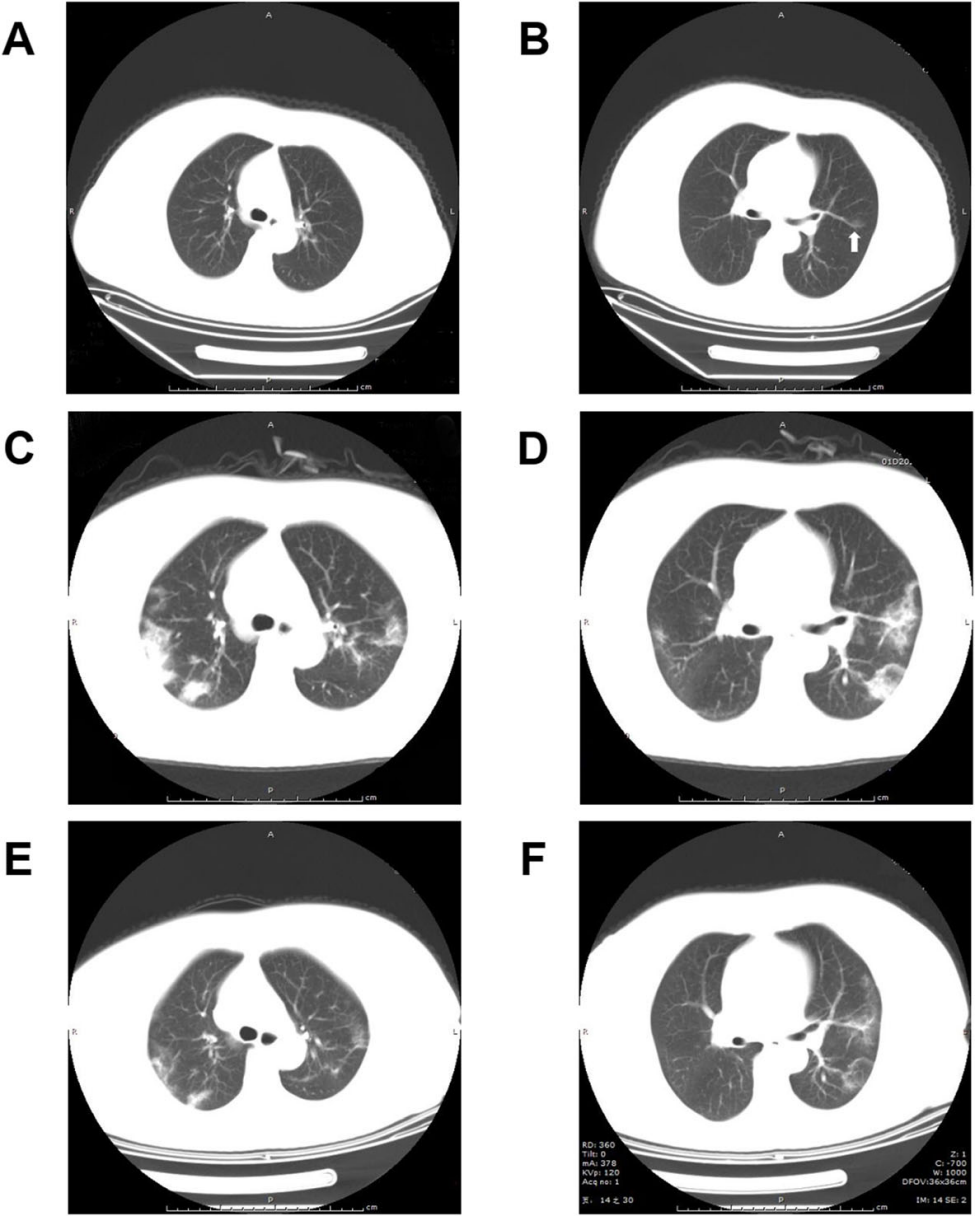
High-resolution computed tomography images. **a** and **b**: Scattered ground-glass opacity lesion was observed (arrow). **c** and **d**: Multiple ground-glass opacity lesions with subpleural distribution were observed in the bilateral lung view, some with consolidation. **e** and **f**: The lesions were significantly absorbed, leaving some blurs

Laboratory results from the time of admission are summarized in Table 1. She got decreased eGFR (53.6 ml/min/1.73m^2^) and deteriorated proteinuria (1.07 g/day, of which, albuminuria was 839 mg/day) when compared to her baseline level. Notably, the patient had mild lymphopenia of 0.86*10^9^/L and increased C reaction protein (CRP) level of 19.6 mg/L. Serologic examinations for hepatitis B virus (HBV), hepatitis C virus (HCV) and human immunodeficiency virus (HIV) were negative. Anti-nuclear antibody, anti-extractable nuclear antigen antibodies, anti-neutrophil cytoplasm antibodies and anti-glomerular basement membrane antibody were negative. Serum immunoglobulin (Ig) A level was slightly increased at 4.71 g/L (reference range: 0.82 g/L-4.53 g/L), whereas IgG, IgM, complement C3 and C4 levels were within the normal range. An ultrasound and a Computed Tomography (CT) examinations for the urinary system were unremarkable. A chest CT scan revealed scattered ground glass opacity (GGO) (Fig. 1a and b).

**Fig. 2:**
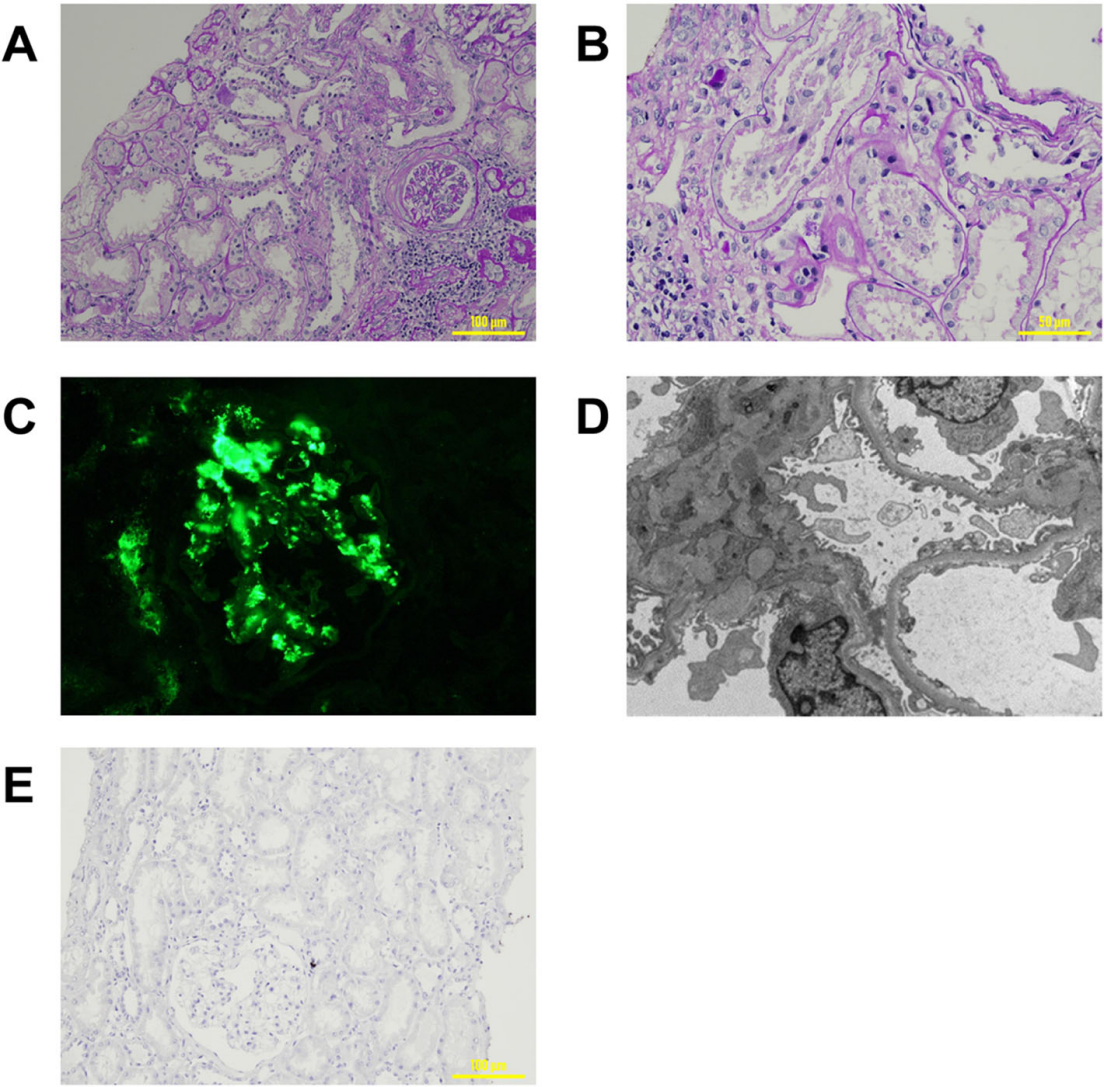
Renal biopsy findings. **a** Glomerulus with a fibrocelluar crescent, adjacent acute tubular injury, as well as associated tubular atrophy/interstitial inflammation (PAS stain; original magnification × 200); **b** Cells debris within the proximal tubular lumen (PAS stain; original magnification × 400); **c** direct immunofluorescence staining with IgA (original magnification × 400); **d** Ultrastructure examination reveals mesangial electron-dense deposits (transmission electron microscopy; original magnification × 2500); **e** Negative immunohistochemistry staining for the S1 spike protein of SARS-CoV-2 (original magnification × 200). PAS: Periodic Acid-Schiff; SARS-CoV-2: severe acute respiratory syndrome coronavirus 2

On admission day 5, a renal biopsy was performed. A total of 16 glomeruli were identified in the tissue submitted for evaluation, 5 of which were completely sclerosed. One glomerulus showed segmental sclerosis and one showed a fibrocellular crescent (Fig. 2a). Focal mild mesangial hypercellularity was identified in rare glomeruli. There was no evidence of significant glomerular inflammation or necrosis. The tubular parenchyma showed moderate interstitial fibrosis associated with nonspecific mononuclear cell inflammatory**.** Intact tubules showed focal acute tubular injurious changes characterized by attenuation of the brush borders and cytoplasmic vacuolization as well as luminal cellular debris (Fig. 2a and b). By immunofluorescence microscopy, the glomeruli showed 2+ granular mesangial staining for IgA (Fig. 2c), C3, kappa and lambda light chains. IgG, IgM and C1q were negative. Electron microscopy (EM) examination revealed mesangial electron dense immune-type deposits. There was no evidence of definitive viral particles (Fig. 2d). A diagnosis of IgA nephropathy with an Oxford score of M_0_E_0_S_1_T_1_C_1_ was rendered.

With the diagnosis of IgA nephropathy, she received valsartan, an angiotensin II receptor blocker (ARB), with an initial dosage of 20 mg per day. She experienced dry cough without fever, dyspnea, diarrhea, myalgia or sore throat on admission day 12. Consequently, we repeated a chest CT scan for her out of caution due to COVID-19. The CT images showed a significant interval progression with a viral pneumonia pattern (Fig. 1c and d). She had progressive chest imaging, however, she had only mild symptoms and her oxygen saturation maintained between 97 and 99%. Her CRP level increased to 36.6 mg/L. A panel of infectious disease screening was initiated including IgM antibodies against nine respiratory pathogens: influenza virus A, influenza virus B, parainfluenza virus, adenovirus, respiratory syncytial virus, pneumonophagous legionella, Q fever rickettsia, mycoplasma pneumoniae and chlamydia pneumoniae, which were all negative. A throat swab specimen tested positive for SARS-CoV-2 later. The frozen renal tissue from biopsy specimens was submitted for reverse transcription-polymerase chain reaction, however, which was tested negative for SARS-CoV-2. Immunohistochemical (IHC) evaluation for the spike protein (40150-R007, Sino Biological, Beijing, China) of SARS-CoV-2 in the kidney was negative as well (Fig. 2e). According to the guideline for COVID-19 issued by the National Health Commission of China [8], she received methylprednisolone (40 mg per day for 3 days) to alleviate her pulmonary inflammation and empirical anti-virus medication (oseltamivir at 75 mg, twice a day for 5 days).

On admission day 17, a follow-up chest CT scan showed a significant improvement (Fig. 1e and f). Laboratory investigations showed stable renal function (Table.1), restored lymphocyte count (1.05*109/L) and decreased CRP level (4.6 mg/L). A repeated throat swab specimen tested negative for SARS-CoV-2. The patient was discharged.

Three months later, the patient remains asymptomatic clinically. A follow-up investigation revealed positive IgG and the IgM antibody against SARS-CoV-2. Her eGFR and UACR were 74.69 ml/min/1.73m^2^ and 33.61 mg/g respectively; her urine erythrocyte was 28.3 cells/μl (her baseline level). The valsartan dosage was titrated to 40 mg OD for optimizing her blood pressure control.

## Discussion and conclusions

The spike protein of SARS-CoV-2 uses ACE2 as a receptor to targeted cells [1]. ACE2 is broadly expressed in human organs especially in the apical brush borders of the proximal tubules and to a less extent in the podocytes in kidneys [9]. This finding raised interest over the relationship between ACE2 and COVID-19. Histological findings from postmortem specimens confirmed the deposition of viral components (e.g. spike protein) in renal tissue and virus-like particles within epithelial cells [10]. Moreover, Pan et al. suggests that kidney has a predisposition to COVID-19 due to ACE2 expression [11]. A recent study from our group reveals that up to 43.9% of patients exhibited renal impairment including abnormal urinalysis and acute kidney injury (AKI) [12]. However, whether these abnormalities are directly caused by the virus infection remains unclear.

The flu-like symptoms, lymphopenia and chest CT image with GGO of the current patient presented prior to the biopsy, although she did not have fever or dyspnea and denied exposure to the Huanan Sea Food Market (the presumptive epidemic origin of the Wuhan City [13]). The sequence of events strongly suggests that the patient got SARS-CoV-2 infection before renal biopsy. Here, the renal biopsy shows acute tubular injury. Although it is mild and focal, it is important to identify whether these changes are directly related to COVID-19. IHC examination for SARS-CoV-2 antigen was negative; viral particles were not identified from EM and nucleic testing in renal tissue was negative as well. As a result, we favor that the acute tubular injury is unlikely to be a direct consequence from COVID-19. However, we cannot completely exclude the possibility that the viral load was too low to be detectable as the biopsy was performed only 8 days from symptoms onset. Recent renal biopsy investigations in patients with COVID-19 presented with acute kidney injury and proteinuria have identified that acute tubular injury was a common histological finding [14–16] and the absence of viral particles did not exclude kidney involvement in COVID-19 [15]. For this reason, the acute renal injury of our patient could be attributed to, at least, in part, SARS-CoV-2 infection.

IgA nephropathy is the most common primary glomerular disease in the world. It is accountable for up to 54.3% of biopsy-proven primary glomerulonephritis in China [17]. Our patient has the usual presentation of IgA nephropathy with gross hematuria and associated reversible AKI, which is frequently seen in patients after bacterial or viral upper respiratory infection [17]. Hematuria-related AKI is common in patients with IgA nephropathy who are older than 65 years [18], and it has been increasingly observed that gross hematuria-associated AKI is reversible when hematuria resolves [19, 20].

Viral infections may exacerbate glomerular disease, such as immune-complex-mediated glomerulopathies related to HCV infection and collapsing glomerulopathy (CG) related to HIV infection [21]. Importantly, recent studies show that patients with COVID-19 who carried the APOL1 gene risk variant developed CG, which raise the possibility that COVID-19 can induce glomerular disease [22]. To date, these morphological changes have not been reported in Chinese patients. However, a longer follow-up period for all patients who have recovered from COVID-19 as well as renal biopsy for patients with persistent proteinuria and/or renal injury may be warranted.

In summary, we reported a patient with COVID-19 with IgA nephropathy. Although the evidence for early viral entry into the kidney is absent, COVID-19 can act as a trigger for exacerbating IgA nephropathy. Renal abnormalities in patients with COVID-19 could be the first presentation of their underlying glomerular disease; if a patient with presumably underlying glomerular nephropathies develops a deterioration of kidney disease during COVID-19, a kidney biopsy may be indicated to exclude rapidly progressive glomerular nephropathies. Further studies are needed for a better understanding of COVID-19, including its effects in kidney. And it is important to consider the underlying glomerular disease when dealing with renal abnormalities in patients with COVID-19.

## Data Availability

If required, the relevant material can be provided by corresponding author on reasonable request.

## Abbreviations

COVID-19: Coronavirus Disease 2019
SARS-Cov-2: Severe acute respiratory syndrome coronavirus 2
CKD: Chronic kidney disease
eGFR: Estimated glomerular filtration rate
HBV: Hepatitis B virus
HCV: Hepatitis C virus
HIV: Human immunodeficiency virus
Ig: Immunoglobulin
CT: Computed tomography
GGO: Ground glass opacity
ARB: Angiotensin II receptor blocker
AKI: Acute kidney injury
CG: Collapsing glomerulopathy
